# Adjusting products with compensatory elements using a digital twin: Model and methodology

**DOI:** 10.1371/journal.pone.0279988

**Published:** 2023-01-03

**Authors:** Jaromir Konecny, Michaela Bailova, Petr Beremlijski, Michal Prauzek, Radek Martinek

**Affiliations:** 1 Department of Cybernetics and Biomedical Engineering, VSB - Technical University of Ostrava, Ostrava-Poruba, Czech Republic; 2 Department of Applied Mathematics, VSB - Technical University of Ostrava, Ostrava-Poruba, Czech Republic; University of Zaragoza, SPAIN

## Abstract

The article presents a novel strategy for reducing the geometric error of a vehicle headlamp equipped with a set of calibration screws, which represents a product assembly. Using a general method for designing and implementing a digital twin, we determined the optimal configuration for a compensatory element that minimizes the total geometric error. Formulated as a problem of constrained minimization, we solved the error using the gradient method and the Broyden–Fletcher–Goldfarb–Shanno method. Products are automatically adjusted according to this optimal setting during the manufacturing process. The results of this novel method indicate that all points can be aligned when the non-individual calibration satifies a geometrical specification of 92%. The digital twin approach was compared to the manufacturing process on 84,055 samples. Overall, 98.19% of the samples were perfectly aligned.

## Introduction

As industrial fields diversify and emerge, customers are placing greater demands on producers to continually improve manufacturing processes [1]. Improving manufacturing today requires significant transformation in many processes and the application of new and modern strategies, for example the conceptual changes delivered by Industry 4.0. The article describes a novel process for adjusting calibration screws in a vehicle headlamp. The target product is protected under a Non-Disclosure Agreement, therefore the paper describes a headlamp model with general parameters only instead of the specified headlamp model. All the experiments presented in this paper applied a general kinematic model to suitably illustrate the calibration method.

The target product has a defined set of testing points on its surface. These are used to verify whether the customer’s requirements for accuracy are satisfied. Equipped with compensating elements in the form of calibration screws, the product allows minor adjustments to its fixing. During production, the calibration screws must be set so that the resulting geometric error at the testing points is minimized.

In the traditional approach, one produced part per day is taken from a production line and precision adjusted manually by an operator with a screwdriver and measured with a coordinate measuring machine (CMM). The same setting for the calibration screws is then applied to every part manufactured on that day, and consequently the geometric error in each part, in theory, is identical. Quality control applies a statistical process control (SPC) method to calculate the process capability index, which is a statistical measurement that represents the ability to make a product within the specification tolerances (see [2]). The traditional method for calibrating the product is time-consuming, and the calibration screws could also not always be set individually.

The main contributions in this paper are as follows:

1. A novel method for automatically adjusting the compensatory elements of individual headlamps directly in the manufacturing process.
2. A practical configuration for a digital twin to geometrically calibrate assemblies that use adjustable compensatory elements measured directly for accuracy.
3. A comparison of two optimization algorithms (gradient method and Broyden–Fletcher–Goldfarb–Shanno) used on the digital twin.

The challenges inherent in obtaining optimal adjustments calls for the use of a digital twin and to simulate the adjustment process virtually for specific calibration screw settings. This approach allows minimization of the locally Lipschitz continuous composite cost function, which in our case is continuously differentiable and subject to inequality constraints. For this purpose, we can use a method suitable for smooth, unconstrained optimization, for example first-order or second-order methods, where the inequality constraints are subject to a quadratic penalty. The solution proposed in this paper applied the gradient method (first-order method) and Broyden–Fletcher–Goldfarb–Shanno (BFGS) algorithm (second-order method) [3–7].

**Fig 1:**
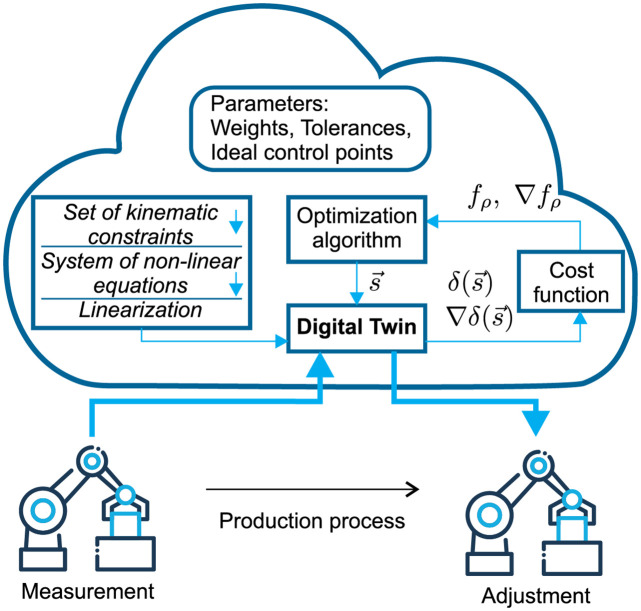
Block diagram of the proposed method.

Fig 1 shows a block diagram of the optimal adjustment procedure using a digital twin. First, an assembled part is measured during the manufacturing process, and then the set of measured points is transferred to a digital twin to simulate the actual geometric parameters. An optimization algorithm then estimates the calibration screw settings and transfers this information to the digital twin. The digital twin thus enables virtual adjustment and calculates the position of the control points, which are subsequently passed to a cost function.

In this case, the cost function consists of two separate functions: inner and outer. The inner function is formulated as an implicit nonlinear vector function that describes the product’s kinematic model. This implicit function assigns the testing point locations $\delta (\vec{s})$ to the arbitrary locations of the calibration screws $\vec{s}$. The outer function is a sum of two functions: the first function evaluates the quality of the adjustment to the product, the second is a quadratic penalty function which penalizes any violation of inequality constraints. The inequality constraints ensure that the set of prescribed limits (tolerances) for testing point locations is not exceeded.

To solve the optimization problem, we applied the gradient method and BFGS algorithm. The optimization algorithm provides a calibration screw setting for the minimum total geometric error and transfers it to the production line. The product is subsequently adjusted according this optimal setting with electronic screwdrivers.

The remainder of the paper is organized as follows. *Related works* provides an overview of the state of the art. In *Methods*, we mathematically describe the gradient method, BGFS algorithm and quadratic penalty method. The *Models* section presents the mass, digital twin and optimization models. *Experiment* describes the experimental procedure. In *Results*, we report the experimental results, followed by a discussion of these results in the *Discussion* section. Finally, we assess the paper’s body of knowledge and outline options for future work in the *Conclusion*.

## Related works

Most automotive parts are manufactured on production lines. Generally, they should be manufactured very precisely according to a geometrical specification to facilitate the assembly process. To fulfil quality control standards [8], all products or selected samples must be measured. The most accurate measurements are done with a CMM [9]. Unfortunately, these machines have several limitations, such as limited dimension range and a long measurement cycle [10]. Especially Considering the long measurement cycle, CMMs are especially unsuitable for checking all aspects on a part. A promising method for measuring parts is inline measurement [11], performed directly on the production line.

Quality control checks apply the Geometrical Product Specifications (GPS) defined by the International Organization for Standardization (ISO). GPS provides the exact definitions for the geometry of components in a product so that producing technical drawings, programming measurement instruments, and estimating measurement uncertainties are unambiguous. GPS characterizes the duality principle by defining the non-ideal surface model of a work piece and defining the verification model according to the verification process executed by the person who inspects the manufactured work piece [12]. The difference between the models indicates a measurement uncertainty [13].

Quality control itself does not increase the manufacturing precision, it only estimates a total geometric error. Research challenges lie in improving production processes for more precise manufacturing. Several options are available generally to increase product precision according to GPS. The first option is to calibrate a production line and its robot manipulators using a CMM and the method presented in [14]. Another option for calibrating robot manipulators is using af laser tracker [15]. This method is suitable for a machine tool which can be modelled as serial manipulator with individual axes behaving as rigid bodies with six degrees of freedom.

The second option is to equip a product with compensating elements such as adjustment screws. This allows the adjustment of fixing points in assembled parts to reduce geometric error. Compensating elements such as these are provided by the Böllhof company [16]. Using special mechanical fixing screws, Böllhoff’s Flexitol solution permits manual and automated infinitely variable tolerance compensation.

The modern approach to maintaining individual production is a digital twin [17]. The term *digital twin* has gained popularity recently in academic and industrial circles [18]. A digital twin is a set of virtual information constructs which fully describe the possible or actual physical manufactured product from the micro-atomic level to the macro-geometric level [19, 20]. At its optimum, any information that could be obtained by inspecting a physically manufactured product can be derived from its digital twin [19]. In the study presented here, the principle of a digital twin was used to obtain the highest geometric quality for the self-adjusting smart assembly line described in [21].

Table 1 summarizes the works related to the proposed solution. The literature review suggests that the commonly applied approach is calibration of the production line using robot manipulators to achieve higher quality products. Interestingly, quality control is never discussed in any of the reviewed literature. The proposed solution uses Böllhoff Flexitol fixing screws [16] to adjust the fixing points in a product and thereby decrease the total geometric error. Inline measurement allows each part to be measured, a procedure which has a significant impact on the Process Capability Index [25]. Instead of using SPC, the Process Capability Index can be evaluated with each part.

**Table 1:** Summary of related works.

Source	Key parameters
Wang et al. [14]	provides production line calibration requires a CMM
Montavon et al. [15]	provides production line calibration requires a laser tracker modelled as a serial manipulator
Pan et al. [22]	provides production line calibration requires cameras or laser sensors extensive image processing
Yin et al. [23]	provides parallel robot manipulator calibration approach based on screw theory to determine identifiable error parameters
Aderiani et al. [24]	individualized locator adjustments digital twin approach improvement of up to 81% in the geometric variation improvement of 78% in the component’s mean deviation
Proposed solution	provides individual product calibration uses Böllhoff Flexitol direct inline measurement measurement and automated calibration on every part

## Methods

This section investigates the following type of unconstrained problem:

$$\begin{array}{c} \underset{x\in R^{n}}{\text{min}}f\left(x\right), \end{array}$$ (1)

where $f:R^{n}\to R$ is a continuously differentiable function in Ω.

Let us apply the following notation: *x*^*T*^ denotes the transpose of the column vector *x* and *E* denotes the identity matrix.

To solve the problem (1), we introduce two iterative optimization algorithms, starting with the gradient method, which represents a first-order method, then a second-order method called the Broyden-Fletcher-Goldfarb-Shanno (BFGS) algorithm. Both methods need a value of *f* and a gradient of *f* at every given point *x*.

### Gradient method

First, let us attempt to solve the problem (1) using a descent direction to reduce the value of *f*.

The gradient method is a special variant of the descent direction method based on the observation that *f* decreases the quickest if we follow the direction of the opposite gradient. For more detailed information about the method, see [3, 4]. The method is still widely used across many scientific disciplines, see for example [26, 27].

We start with an initial guess *x*_0_. The algorithm produces a sequence of iterations *x*_1_, *x*_2_, …. In every iteration *x*_*k*_, the algorithm evaluates ∇*f*(*x*_*k*_) and finds the descent direction $d\in R^{n}$, in the following manner:

$$\begin{array}{c} d=-\frac{\nabla f(x_{k})}{∥\nabla f(x_{k})∥}. \end{array}$$ (2)

A new iteration is acquired by moving *x*_*k*_ in the direction *d*, i.e.

$$\begin{array}{c} x_{k+1}=x_{k}+t^{*}d,\;\;\;t^{*}>0. \end{array}$$ (3)

The algorithm continues searching until ‖*x*_*k*+1_ − *x*_*k*_‖ ≤ *ε*, where *ε* is a given precision.

The pseudocode of the gradient method is described in Algorithm 1.

**Algorithm 1:** Gradient method *f*, *x*_0_

1: take $x_{0}\in R^{n}$, set $d=-\frac{\nabla f(x_{0})}{∥\nabla f(x_{0})∥}$, *k* = 0

2: **while** ‖∇*f*(*x*_*k*_)‖ ≥ *ε* **do**

3:  take t* > 0 {we consider t* that guarantees the reduction of *f*}

4:  *x*_*k*+1_ = *x*_*k*_ + t*d_*k*_

5:  $d_{k+1}=-\frac{\nabla f(x_{k+1})}{∥\nabla f(x_{k+1})∥}$

6:  *k* = *k* + 1

7: **end while**

8: set *x* = *x*_*k*_

To find the optimal step length t*, we use the descent direction and compute a local minimum of *f* in the direction *d*, where

$$\begin{array}{c} t^{*}=\text{arg}\underset{t\in R,t>0}{\text{min}}f\left(x_{k}+td\right). \end{array}$$ (4)

For practical computation, it is easier to turn (4) into a constrained problem:

$$\begin{array}{c} t^{*}=\text{arg}\underset{t\in R,t\in [0,t_{\text{max}}]}{\text{min}}f\left(x_{k}+td\right), \end{array}$$

where *t*_max_ is sufficiently large.

For the one-dimensional constrained optimization of *f* with respect to *t*, we use the Golden Section method (see [3]).

### BFGS algorithm

The Broyden-Fletcher-Goldfarb-Shanno (BFGS) algorithm introduced here is based on Newton’s method. It is a second order method using a quadratic model of *f* in the neighbourhood of *x*_*k*_, i.e.

$$\begin{array}{c} f\left(x\right)\approx m_{k}\left(x\right)=f\left(x_{k}\right)+{\left(x-x_{k}\right)}^{T}\nabla f\left(x_{k}\right)+\\ +\frac{1}{2}{\left(x-x_{k}\right)}^{T}\nabla^{2}f\left(x_{k}\right)\left(x-x_{k}\right). \end{array}$$ (5)

We want to find a minimum of *m*_*k*_, i.e. find *x* as a stationary point of *m*_*k*_,

$$\begin{array}{c} \nabla m_{k}\left(x\right)=0, \end{array}$$

which means

$$\begin{array}{c} \nabla f\left(x_{k}\right)+\nabla^{2}f\left(x_{k}\right)\left(x-x_{k}\right)=0. \end{array}$$ (6)

The stationary point is labelled *x*_*k*+1_. From (6), we obtain an expression of Newton’s iteration

$$\begin{array}{c} x_{k+1}=x_{k}-\nabla^{2}f{\left(x_{k}\right)}^{-1}\nabla f\left(x_{k}\right), \end{array}$$ (7)

where

$$\begin{array}{c} \nabla^{2}f\left(x_{k}\right)=(\begin{array}{ccc} \frac{\partial^{2}f\left(x_{k}\right)}{\partial x_{1}\partial x_{2}} & \cdots & \frac{\partial^{2}f\left(x_{k}\right)}{\partial x_{1}\partial x_{n}}\\ \vdots & \ddots & \vdots \\ \frac{\partial^{2}f\left(x_{k}\right)}{\partial x_{n}\partial x_{2}} & \cdots & \frac{\partial^{2}f\left(x_{k}\right)}{\partial x_{n}\partial x_{n}} \end{array}) \end{array}$$

is the Hessian of *f*. We can denote

$$\begin{array}{c} d_{k}=\nabla^{2}f{\left(x_{k}\right)}^{-1}\nabla f\left(x_{k}\right). \end{array}$$

Moreover, if the Hessian is a positive definite matrix, i.e.

$$\begin{array}{c} \forall x\in R^{n},x\neq \vec{0}:x^{T}\nabla^{2}f\left(x_{k}\right)x>0, \end{array}$$

and ∇*f*(*x*_*k*_) ≠ 0, it is true that *d*_*k*_ is a descent direction of *f*.

The BFGS algorithm is introduced here is a method which uses Newton’s step. In contrast to Newton’s method, the Hessian is approximated using the results of the previous iterations, known as a Quasi-Newton’s method. A detailed introduction of the method can be found in [4, 7]. Even today, the algorithm is used in many scientific fields, see for example [28, 29].

The approximation *H*_*k*_ of the Hessian ∇^2^*f*(*x*_*k*_) is computed as follows:

$$\begin{array}{c} H_{k}=H_{k-1}+\frac{yy^{T}}{y^{T}s}-\frac{H_{k-1}ss^{t}H_{k-1}}{s^{T}H_{k-1}s}, \end{array}$$ (8)

where *y* = *x*_*k*_ − *x*_*k*−1_ and *s* = ∇*f*(*x*_*k*_) − ∇*f*(*x*_*k*−1_). The pseudocode of the BFGS algorithm is described in Algorithm 2.

**Algorithm 2:** BFGS *f*, *x*_0_

1: take *ε* > 0, $x_{0}\in R^{n}$

2: set *H*_0_ = *E*, *k* = 1

3: **while** ‖∇*f*(*x*_*k*_)‖ ≥ *ε* **do**

4:  *x*_*k*_ = *x*_*k*−1_ − (*H*_*k*−1_)^−1^*g*_*k*−1_

5:  *y* = *x*_*k*_ − *x*_*k*−1_

6:  *s* = ∇*f*(*x*_*k*_) − ∇*f*(*x*_*k*−1_)

7:  **if** *y*^*T*^*s* > 0 **then** {if the Hessian is positive definite}

8:   $H_{k}=H_{k-1}+\frac{yy^{T}}{y^{T}s}-\frac{H_{k-1}ss^{t}H_{k-1}}{s^{T}H_{k-1}s}$

9:  **else**

10:   *H*_*k*_ = *H*_*k*−1_

11:  **end if**

12:  *k* = *k* + 1

13: **end while**

14: set *x* = *x*^*k*^

### Quadratic penalty method

For the purposes here, we need to minimize *f*(*x*) subject to inequality constraints. We solve the following constrained problem:

$$\begin{array}{cc} & \underset{x\in \Omega }{\text{min}}\;f\left(x\right),\\ & \Omega =\{x\in R^{n}:g\left(x\right)\leq \vec{0}\},\\ & g:R^{n}\to R^{r},\;r<n, \end{array}$$ (9)

where *f* and *g* are continuously differentiable. To apply the algorithms introduced above, we need to approximate our constrained problem (9) with an unconstrained problem. We apply a Quadratic Penalty method to approximate the original solution with a solution to the following:

$$\begin{array}{cc} & \underset{x\in R^{n}}{\text{min}}f_{\rho }\left(x\right),\\ & f_{\rho }\left(x\right)≔f\left(x\right)+\frac{1}{2}\rho \alpha {\left(x\right)}^{T}\alpha \left(x\right),\\ & \alpha_{i}\left(x\right)≔\max\left\{g_{i}\left(x\right),0\right\}. \end{array}$$ (10)

For large *ρ*, the approximate solution cannot be far from the solution of the original problem. Furthermore, for *ρ* → ∞, the solution of (10) is also a solution of (9).

## Models

This section introduces a mathematical model for the digital twin and describes the mass, kinematic model and optimization models in detail.

To indicate points, we use capital letters and two types of index. The upper index refers to the index of the point, the lower index represents the coordinate. The vector which describes the positions of the calibration screws is denoted $\vec{s}$.

### Mass

Headlamps must fulfil GPS and consist of a housing and glass glued together. Gluing is a possible source of imprecision if the glass is inaccurately positioned. To check the quality, we measure the the geometric dimensions. The product’s specification defines a set of test points and vectors. Each vector of the set defines the direction of a measuring sensor.

The specification also defines the constraint points fixed to or supported in the product. The set of test points and vectors and set of constrained points provide the basis for the digital twin. In this study, we define the following types of fixing:

- Calibration screw—this fixation type has three degrees of rotational freedom. It can be adjusted (and fixed) along a specific vector, and the remaining perpendicular directions provide the next two degrees of freedom.
- Spike—this fixation type has three degrees of rotational freedom and another degree of freedom in a specific vector (straight line). The spike is a point to line constraint.
- Ball bearing—this fixation has only three degrees of rotational freedom. Other movements are not possible.
- A propped point—this fixation type has three degrees of rotational freedom. Movement is restricted to a plane, or specifically, the propped point slides along a plane surface.

### Digital twin

The following section mathematically describes the constraint points. Using a digital twin, we simulated kinematic behavior. The digital twin allows adjustment of the the screws in virtual space and substitutes an approach involving a complicated procedure by an operator who attempts to find an optimal calibration screw setting with a screwdriver and CMM. This section describes the mathematical formulation of the kinematic model which represents the digital twin.

The main idea behind this approach is the assumption that a headlamp is a rigid body and that if we consider the distances between pairs of points and other aspects given by the constraint points, we can obtain a system of equations to describe the object.

We assume that the distances remain constant. For a given $\vec{s}$ representing the position of the calibration screws, we can compute the shift *δ* at all test points as a solution of a system of nonlinear equations. The system is represented by the following expression:

$$\begin{array}{c} F(\delta \left(\vec{s}\right),\vec{s})=0, \end{array}$$

where *F* is a vector function, $F:R^{q}\to R^{q}$.

For the small values of $\vec{s}$ that we are interested in, the system *F* is nearly linear. We can therefore obtain an approximation of *δ* in $\vec{s}$ as a solution of

$$\begin{array}{c} \delta \left(\vec{s}\right)=\delta_{0}-J_{F}^{-1}\left(\delta_{0},\vec{s}\right)F\left(\delta_{0},\vec{s}\right), \end{array}$$

where *J*_*F*_ is a Jacobian matrix of *F* with respect to *δ*, i.e.

$$\begin{array}{c} J_{F}=(\begin{array}{ccc} \frac{\partial F_{1}}{\partial \delta_{1}} & \ldots & \frac{\partial F_{1}}{\partial \delta_{q}}\\ \vdots & \ddots & \vdots \\ \frac{\partial F_{q}}{\partial \delta_{1}} & \ldots & \frac{\partial F_{q}}{\partial \delta_{q}} \end{array}). \end{array}$$

Furthermore, we can take *δ*_0_ = 0 and obtain

$$\begin{array}{c} \delta \left(\vec{s}\right)=-J_{F}^{-1}\left(0,\vec{s}\right)F\left(0,\vec{s}\right). \end{array}$$ (11)

Let us now describe mathematically the constraint and the test point shifts and specify the elements of *δ*.

- Calibration screw *S*—in general, *S* is screwed in a direction parallel to one of the axes. Movement in the other two coordinates is free. For example, if we decide to screw in the *z* direction, the location of *S* after transformation is described as
  $$\begin{array}{c} S^{\prime }=\left[S_{1},S_{2},S_{3}\right]+\left(\delta_{1}^{S},\delta_{2}^{S},z\right). \end{array}$$
- Spike *T*—this type of point shifts only in the prescribed direction $n^{T}=\left(n_{1}^{T},n_{2}^{T},n_{3}^{T}\right)$. The position after transformation is described as
  $$\begin{array}{c} T^{\prime }=\left[T_{1},T_{2},T_{3}\right]+\delta^{T}\left(n_{1}^{T},n_{2}^{T},n_{3}^{T}\right). \end{array}$$
- Ball bearing (or fixed point) *K*—this type of point has no degrees of freedom, and its position after transformation remains the same
  $$\begin{array}{c} K^{\prime }=K=\left[K_{1},K_{2},K_{3}\right]. \end{array}$$
- Propped point *P*^*d*^—this point must belong to a plane *ρ* defined by a given normal vector $n^{\rho }=\left(n_{1}^{\rho },n_{2}^{\rho },n_{3}^{\rho }\right)$. For (*P*^*d*^)′ ∈ *τ*, it must be true that
  $$\begin{array}{c} n_{1}^{\rho }\left(P_{1}^{d}+\delta_{1}^{P^{d}}\right)+n_{2}^{\rho }P_{2}^{d}+\delta_{2}^{P^{d}})+n_{3}^{\rho }\left(P_{3}^{d}+\delta_{3}^{P^{d}}\right)+d=0, \end{array}$$
  where
  $$\begin{array}{c} d=-n_{1}^{\rho }P_{1}^{d}-n_{2}^{\rho }P_{2}^{d}-n_{3}^{\rho }P_{3}^{d}. \end{array}$$
- Test point *P*^*i*^—the point has generally no defined limits in terms of shift or rotation. Its position after transformation is described as
  $$\begin{array}{c} P^{\prime i}=P^{i}+\left[\delta_{1}^{i},\delta_{2}^{i},\delta_{3}^{i}\right]. \end{array}$$
  The test point positions and the shifts *δ*^*i*^ play a key role in the optimization problem introduced in this article.

### Optimization model

The aim of the method is to find the optimal configuration of the calibration screws *S*^1^, *S*^2^, …, where the distances between test points *P*^*i*^ and their prescribed locations $P_{\text{prec}}^{i}$ are minimal. Regarding the technical aspects, we cannot use the Euclidean norm to compute $∥P^{i}P_{\text{prec}}^{i}∥$ and must develop a more complex method.

For each test point, we have a sensor *B* with an approach vector $\vec{m}={\vec{BP}}_{\text{prec}}^{i}$. The point *B* and vector $\vec{m}$ determine the line *p*.

We construct a plane *ρ* perpendicular to *p* containing the point *P*^*i*^ and find the point $P_{p\rho }^{i}$ as an intersection of *p* and *ρ*. We then define the desired distance as

$$\begin{array}{c} ∥P^{i}P_{\text{prec}}^{i}{∥}_{B}≔∥P_{p\rho }^{i}P_{prec}^{i}∥. \end{array}$$

**Fig 2:**
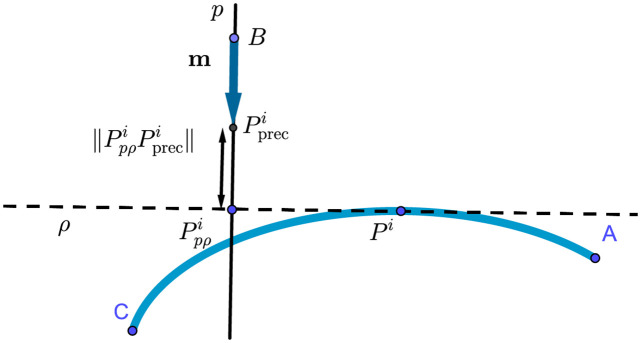
Measuring the distance between *P*^*i*^ and $P_{\text{prec}}^{i}$.

This is illustrated in Fig 2.

We solve the following constrained optimization problem

$$\begin{array}{cc} & \underset{\vec{s}\in R^{n}}{\text{min}}f\left(\vec{s}\right)≔\\ & ≔\underset{\vec{s}\in R^{n}}{\text{min}}\sum_{i=1}^{k}(\sum_{j=1}^{3}w_{i}{\left(P_{j}^{i}-P_{prec\;j}^{i}+\delta_{j}^{i}\left(\vec{s}\right)\right)}^{2}) \end{array}$$ (12)

with the set of constraints related to the distances $∥P^{i}P_{\text{prec}}^{i}{∥}_{B}$,

$$\begin{array}{c} \sum_{j=1}^{3}{\left(m_{ij}t_{i}(\vec{s})\right)}^{2}<{\text{tol}}_{i}^{2},\;\;\;\;\forall i\in \left\{1,\ldots ,k\right\}, \end{array}$$

where

$$\begin{array}{c} t^{i}\left(\vec{s}\right)=\sum_{j=1}^{3}\frac{\left(m_{j}^{i}\right)}{∥m^{i}{∥}^{2}}\left(P_{j}^{i}-P_{prec\;j}^{i}+\delta_{j}^{i}\left(\vec{s}\right)\right), \end{array}$$

$\delta^{i}\left(\vec{s}\right)$ is a vector describing the shift of the point *P*^*i*^ depending on $\vec{s}$, and *w*_*i*_ represents the weight of *P*^*i*^. In other words, the value *w*_*i*_ indicates the importance of aligning *P*^*i*^ near the ideal position. Let us specify that our task is to solve the problem of quadratic programming with nonlinear inequality constraints.

We apply the Quadratic Penalty method introduced above and transform (12) into an unconstrained problem

$$\begin{array}{cc} & \underset{\vec{s}\in R^{n}}{\text{min}}\;f_{\rho }\left(\vec{s}\right)≔\\ & ≔\underset{\vec{s}}{\text{min}}\sum_{i=1}^{k}(\sum_{j=1}^{3}w_{i}{\left(P_{j}^{i}-P_{prec\;j}^{i}+\delta_{j}^{i}\left(\vec{s}\right)\right)}^{2})+\frac{1}{2}\rho \alpha^{\prime }\left(\vec{s}\right)\alpha \left(\vec{s}\right), \end{array}$$ (13)

where

$$\begin{array}{c} \alpha_{i}\left(\vec{s}\right)≔\max\{\sum_{j=1}^{3}{\left(m_{ij}t_{i}(\vec{s})\right)}^{2}-{\text{tol}}_{i}^{2},0\}. \end{array}$$

This type of problem can be solved using either of the optimization methods introduced above. Considering the properties of the cost function, it can also be shown that the gradient method converges linearly to the minimum. The convergence of the BFGS algorithm is superlinear.

## Experiment

In this section, we provide an example of a design for a digital twin and formulate the kinematic model of a headlamp and application of the optimization algorithm.

### Data

For the numerical experiments, we considered a model with the following specification:

- Two calibration screws *S*^1^ and *S*^2^—both are screwed in the *z* direction, while movement in the other directions is free.
- A spike *T*—this point shifts only in the direction defined by a vector ${\vec{n}}^{T}$.
- Two propped points *P*^*d*1^ and *P*^*d*2^—both points must remain in the given planes defined by normals $n^{\rho_{1}}$ and $n^{\rho_{2}}$.
- The headlamp does not contain any ball bearings.

We also considered the set of test points *P*^*i*^, *i* ∈ {1, …*n*} that we would like to align to an optimal position.

If we consider all the model’s properties, we obtain a vector of unknowns:

$$\begin{array}{c} \begin{array}{c} \delta =(\delta_{Pd_{1}1},\delta_{Pd_{1}2},\delta_{Pd_{1}3},\delta_{Pd_{2}1},\delta_{Pd_{2}2},\delta_{Pd_{2}3},\delta_{T},\\ \delta_{S_{1}^{1}},\delta_{S_{2}^{1}},\delta_{S_{1}^{2}},\delta_{S_{2}^{2}},\delta_{P_{i}1},\delta_{P_{i}2},\delta_{P_{i}3}{,\ldots )}^{T}, \end{array} \end{array}$$ (14)

where the first 11 elements relate to shifts in the constraint points and the remaining 3*n* elements describe the transformation of the test points needed to evaluate the cost function. It must be true that the distance between each pair of points remains constant.

For a given $\vec{s}=\left(s_{1},s_{2}\right)$, let us formulate the system of equations which define our kinematic model. First, we take into account the distances between the test points and constraint points; a prime symbol denotes the points after transformation. We thus obtain *n* equations in the following form:

$$\begin{array}{c} ∥P^{\prime i}T^{\prime }∥=d_{T}^{i}≔∥P^{i}T∥\;\;\;\forall i\in \left\{1,\ldots ,n\right\}. \end{array}$$ (15)

The previous equations can be modified to

$$\begin{array}{c} ∥P^{\prime i}T^{\prime }{∥}^{2}-{\left(d_{T}^{i}\right)}^{2}=0\;\;\;\forall i\in \left\{1,\ldots ,n\right\}. \end{array}$$ (16)

Consequently, we can focus on a combination of test points and both calibration screws and obtain another 2*n* equations:

$$\begin{array}{c} ∥P^{\prime i}S^{\prime 1}∥=d_{S^{1}}^{i}≔∥P^{i}S^{1}∥\;\;\;\forall i\in \left\{1,\ldots ,n\right\}, \end{array}$$ (17)

$$\begin{array}{c} ∥P^{\prime i}S^{\prime 2}∥=d_{S^{2}}^{i}≔∥P^{i}S^{2}∥\;\;\;\forall i\in \left\{1,\ldots ,n\right\}. \end{array}$$ (18)

The last coordinate of *S*^*i*^ is equal to *s*_*i*_, which is given.

The propped points belong to given planes and are described by the following two equations:

$$\begin{array}{c} P^{d1}\in \rho_{1}:\sum_{j=1}^{3}n_{j}^{\rho_{1}}\left(P_{j}^{d1}+\delta_{j}^{d1}\right)-\sum_{j=1}^{3}n_{j}^{\rho_{1}}P_{j}^{d1}=0, \end{array}$$ (19)

$$\begin{array}{c} P^{d2}\in \rho_{2}:\sum_{j=1}^{3}n_{j}^{\rho_{2}}\left(P_{j}^{d2}+\delta_{j}^{d2}\right)-\sum_{j=1}^{3}n_{j}^{\rho_{2}}P_{j}^{d2}=0. \end{array}$$ (20)

The points must also be a constant distance from the spike and both calibration screws. Therefore,

$$\begin{array}{c} ∥P^{\prime dl}T^{\prime }∥=d_{T}^{i}≔∥P^{dl}T∥,\;\;\;\forall l\in \left\{1,2\right\}, \end{array}$$ (21)

$$\begin{array}{c} ∥P^{\prime dl}S^{\prime k}∥=d_{P^{dl}S^{k}}^{i}≔∥P^{dl}S^{k}∥\;\;\;\forall k,l\in \left\{1,2\right\}, \end{array}$$ (22)

and we obtain another 8 equations. The final three equations reflect the distances between pairs of constraint points.

$$\begin{array}{c} ∥T^{\prime }S^{\prime k}∥=d_{TS^{k}}^{i}≔∥TS^{k}∥ \end{array}$$ (23)

$$\begin{array}{c} ∥P^{\prime d1}P^{\prime d2}∥=d^{P^{d}P^{d}}≔∥P^{\prime d1}P^{\prime d2}∥. \end{array}$$ (24)

We can rewrite Eqs (17)–(24) in the same manner as Eq (15).

Finally, we obtain a system of equations that can be written as

$$\begin{array}{c} F(\delta \left(\vec{s}\right),\vec{s})=0, \end{array}$$ (25)

where $F:R^{q}\to R^{q}$, *q* = 3*n* + 11 is a vector function. Each component of this function is defined from Eqs (15), (17)–(24) (e.g., the first component of the vector function *F* is defined by the left side of Eq (16)). As mentioned above, we exploit the system’s approximately linearity for small values of *s*_1_, *s*_2_ and compute *δ* from

$$\begin{array}{c} \delta \left(\vec{s}\right)=-J_{F}^{-1}\left(\delta_{0},\vec{s}\right)F\left(\delta_{0},\vec{s}\right), \end{array}$$ (26)

where *J* is a matrix of the first derivatives of *F* with respect to all variables in *δ*.

### Gradient and BFGS method

The methods introduced here are designed for unconstrained optimization. For each given vector $\vec{s}$, we need to evaluate the cost function (13) and compute the gradient.

The gradient can be computed as follows:

$$\begin{array}{c} \nabla f_{\rho }\left(\vec{s}\right)=\nabla f\left(\vec{s}\right)+\rho \sum_{i=1}^{n}\alpha^{i}\left(\vec{s}\right)\nabla g^{i}\left(\vec{s}\right), \end{array}$$ (27)

where

$$\begin{array}{cc} & \nabla f(\vec{s})=\\ & (\begin{array}{c} \sum_{i=1}^{k}\sum_{j=1}^{3}2w_{i}(P_{j}^{i}-P_{\text{prec}j}^{i}+\delta_{j}^{i}\left(\vec{s}\right))\frac{\partial \delta_{j}^{i}\left(\vec{s}\right)}{\partial s_{1}}\\ \sum_{i=1}^{k}\sum_{j=1}^{3}2w_{i}(P_{j}^{i}-P_{\text{prec}j}^{i}+\delta_{j}^{i}\left(\vec{s}\right))\frac{\partial \delta_{j}^{i}\left(\vec{s}\right)}{\partial s_{2}} \end{array}). \end{array}$$ (28)

The elements of ∇*g* can be calculated in the following manner:

$$\begin{array}{cc} & \nabla g^{i}\left(\vec{s}\right)=2\cdot \\ & \cdot (\begin{array}{c} \sum_{k=1}^{3}\sum_{j=1}^{3}m_{k}^{i}\frac{m_{j}^{i}}{∥m^{i}{∥}^{2}}(P_{j}^{i}+\delta_{j}^{i}\left(\vec{s}\right)-P_{\text{prec}j}^{i})\frac{\partial \delta_{j}^{i}\left(\vec{s}\right)}{\partial s_{1}}\\ \sum_{k=1}^{3}\sum_{j=1}^{3}m_{k}^{i}\frac{m_{j}^{i}}{∥m^{i}{∥}^{2}}(P_{j}^{i}+\delta_{j}^{i}\left(\vec{s}\right)-P_{\text{prec}j}^{i})\frac{\partial \delta_{j}^{i}\left(\vec{s}\right)}{\partial s_{2}} \end{array}). \end{array}$$ (29)

Now we compute $\frac{\partial \delta_{j}^{i}\left(\vec{s}\right)}{\partial s_{1}}$ and $\frac{\partial \delta_{j}^{i}\left(\vec{s}\right)}{\partial s_{2}}$. Returning to (26) and modifying the equation, we obtain

$$\begin{array}{c} J\left(\delta_{0},\vec{s}\right)\delta \left(\vec{s}\right)=-F\left(\delta_{0},\vec{s}\right). \end{array}$$ (30)

If we first differentiate both sides of (30) with respect to *s*_1_, we obtain

$$\begin{array}{c} \frac{\partial J_{F}\left(\delta_{0},\vec{s}\right)}{\partial s_{1}}\delta \left(\vec{s}\right)+J_{F}\left(\delta_{0},\vec{s}\right)\frac{\partial \delta (\vec{s})}{\partial s_{1}}=-\frac{\partial F(\delta_{0},\vec{s})}{\partial s_{1}}. \end{array}$$

After a slight modification, we obtain

$$\begin{array}{c} \frac{\partial \delta (\vec{s})}{\partial s_{1}}=J_{F}^{-1}\left(\delta_{0},\vec{s}\right)(-\frac{\partial J_{F}\left(\delta_{0},\vec{s}\right)}{\partial s_{1}}\delta \left(\vec{s}\right)-\frac{\partial F(\delta_{0},\vec{s})}{\partial s_{1}}). \end{array}$$

Consequently, we can differentiate (30) with respect to the other variable

$$\begin{array}{c} \frac{\partial J_{F}\left(\delta_{0},\vec{s}\right)}{\partial s_{2}}\delta \left(\vec{s}\right)+J_{F}\left(\delta_{0},\vec{s}\right)\frac{\partial \delta (\vec{s})}{\partial s_{2}}=-\frac{\partial F(\delta_{0},\vec{s})}{\partial s_{2}} \end{array}$$

and express $\frac{\partial \delta_{j}^{i}\left(\vec{s}\right)}{\partial s_{2}}$:

$$\begin{array}{c} \frac{\partial \delta (\vec{s})}{\partial s_{2}}=J_{F}^{-1}\left(\delta_{0},\vec{s}\right)(-\frac{\partial J_{F}\left(\delta_{0},\vec{s}\right)}{\partial s_{2}}\delta \left(\vec{s}\right)-\frac{\partial F(\delta_{0},\vec{s})}{\partial s_{2}}). \end{array}$$

### Simple model example

Let us now illustrate the previous relationship which describes the kinematic model of the headlamp and application of the optimization algorithms on a simple model. The model in this example contains only one calibration screw *S*, one spike *T*, one propped point *P*^*d*^, and one test point *P* and its prescribed location *P*_prec_.

The specification in the given model is as follows:

- Calibration screw *S* = [0, 0, 0]—screwed only in the *z* direction, while movement in other directions is fixed.
- Spike *T* = [1, 0, 0]—the point shifts only in the direction ${\vec{n}}^{T}=\left[0,0,1\right]$.
- Propped point *P*^*d*^ = [−1, 0, 1]—the point must remain in the plane *ρ* defined by normal *n*^*ρ*^ = [−1, 0, 0], the movement in the *x* direction is fixed.
- Test point *P* = [0, 0, 1], which we want to adjust to the optimal position *P*_prec_ = [0, 0, 2].

**Fig 3:**
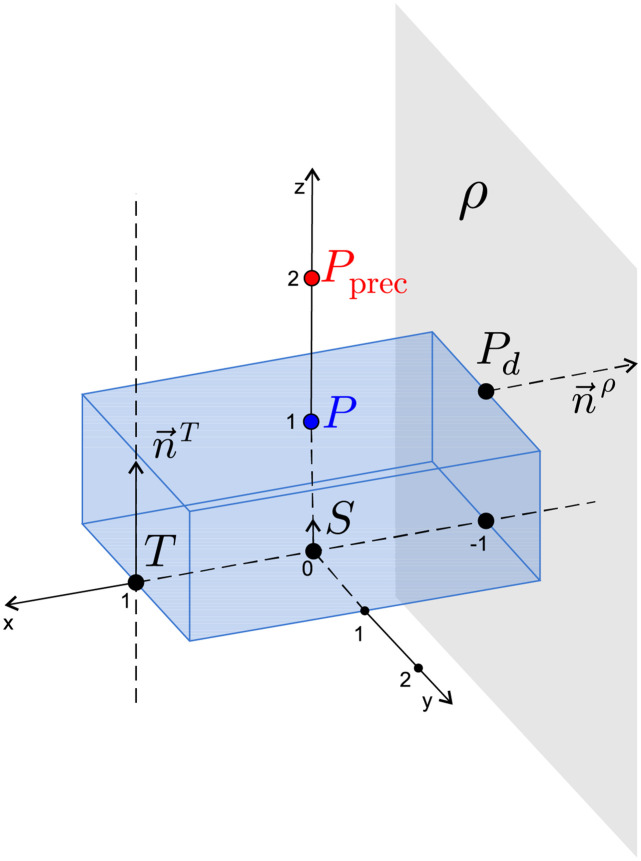
Example of a simple model with one calibration screw.

The model is illustrated in Fig 3.

For this simple model, we obtain the following vector of unknowns:

$$\begin{array}{c} \begin{array}{c} \delta ={\left(\delta_{Pd2},\delta_{Pd3},\delta_{T},\delta_{P_{1}},\delta_{P_{2}},\delta_{P_{3}}\right)}^{T}, \end{array} \end{array}$$ (31)

where the first three unknowns relate to shifts of the constraint points, and the remaining three unknowns describe the transformation of the test point we need for evaluating the cost function.

Let us now describe the kinematic model for this example, obtained in the same manner as the system of Eq (25).

We formulate the system of equations for the model (a prime symbol denotes the points after transformation)

$$\begin{array}{c} ∥P^{\prime }T^{\prime }{∥}^{2}-{∥PT∥}^{2}=0,\;\text{where}\;∥PT∥=2. \end{array}$$ (32)

$$\begin{array}{c} ∥P^{\prime }S^{\prime }{∥}^{2}-{∥PT∥}^{2}=0,\;\text{where}\;∥PS∥=1. \end{array}$$ (33)

$$\begin{array}{c} P^{d}\in \rho :\sum_{j=1}^{3}n_{j}^{\rho }\left(P_{j}^{d}+\delta_{j}^{d}\right)-\sum_{j=1}^{3}n_{j}^{\rho }P_{j}^{d}=0. \end{array}$$ (34)

$$\begin{array}{c} ∥P^{\prime d}T^{\prime }{∥}^{2}-∥P^{d}{T∥}^{2}=0,\;\text{where}\;∥P^{d}T∥=\sqrt{5}. \end{array}$$ (35)

$$\begin{array}{c} ∥P^{\prime d}S^{\prime }{∥}^{2}-∥P^{d}{S∥}^{2}=0,\;\text{where}\;∥P^{d}S∥=\sqrt{2}. \end{array}$$ (36)

$$\begin{array}{c} ∥T^{\prime }S^{\prime }{∥}^{2}-{∥TS∥}^{2}=0,\;\text{where}\;∥TS∥=1. \end{array}$$ (37)

Finally, we obtain a system of equations that can be written as

$$\begin{array}{c} F(\delta \left(\vec{s}\right),\vec{s})=0, \end{array}$$ (38)

where $F:R^{q}\to R^{q}$, *q* = 6 is a vector function. Each component of this function is defined by the left side of Eqs (32)–(37). Eq (38) is solved using the expression in (26).

Now let us consider the optimization problem describing the calibration of screws during headlamp adjustment. For simplicity, in this example, we do not require a given tolerance to be satisfied for the test point P, i.e., we consider only the unconstrained optimization problem.

For this example, we obtain the following optimization problem:

$$\begin{array}{cc} & \underset{\vec{s}\in R}{\text{min}}\sum_{j=1}^{3}w_{i}{\left(P_{j}-P_{prec\;j}+\delta_{j}\left(\vec{s}\right)\right)}^{2}. \end{array}$$ (39)

The analytical solution to the problem is $\vec{s}=1$.

**Algorithm 3:** Description of the calibration procedure

1: A headlamp is placed onto a mounting stand by an operator.

2: A shelf with a stand is transferred to a calibration area.

3: Robotic arms and tactile sensors perform precise measurement.

4: The digital twin calculates optimal calibration screw settings.

5: Automated screwdrivers set the target screw settings.

6: A protocol with estimated tolerances is stored in the database.

**Fig 4:**
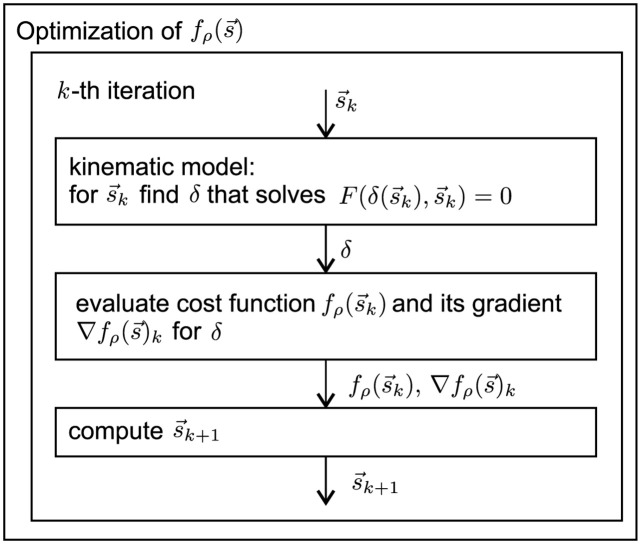
Flowchart of the optimization procedure—*k*-th iteration.

An iteration of the optimization method for headlamp adjustment is explained schematically in Fig 4. Algorithm 3 indicates the entire procedure for the calibration process of one headlamp on a deployed machine.

## Results

**Fig 5:**
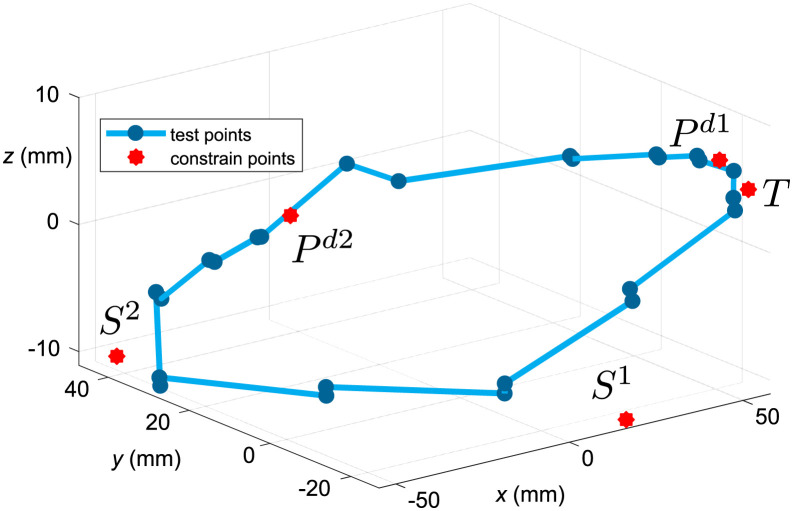
General shape of the model, test points and constraint points.

For the numerical experiments, let us consider the headlamp introduced in the previous section, with two calibration screws *S*^1^ and *S*^2^, two propped points, one spike, and 25 test points. Both *S*^1^ and *S*^2^ can only be screwed in the *z* direction. At every test point, we are given a tolerance, i.e. a maximum permitted distance from the prescribed position. Note that the distance is measured in the ‖.‖_*B*_ norm defined above. Fig 5 shows a sketch of the model. The test points we want to align to the prescribed positions are indicated with blue dots, the constraint points are indicated with red stars.

We simulated real situations and applied both algorithms. By computing the configuration of the calibration screws, a headlamp model can be placed in the desired position to allow evaluation of the distances between the test points and their prescribed positions. All the test point tolerances and shifts are small in relation to the product’s size, guaranteeing that the values of *s*_1_ and *s*_2_ remain sufficiently small.

Starting with an ideal headlamp model, all points are located exactly in the prescribed positions. For any given movement in the calibration screws $\vec{s}=\left(s_{1},s_{2}\right)$, the headlamp model is transformed according to the kinematic model. The optimization algorithm is then applied to move it back into the original position. This type of simulation guarantees that none of the model’s mathematical conditions are violated and provides an excellent opportunity to study the behaviours of the algorithms. It also provides an easy way to verify the output of the algorithm.

We then applied the same conditions as in the previous simulation, but after transformation, slightly adjusted the test points using shifts. This procedure simulated an imperfect manufacturing process or glass imprecisely glued to the housing.

The final simulation modelled an ideal headlamp and moved it into an initial position. Unlike the previous simulation, the product was transformed using general shifts and rotations. The optimization algorithm then calculated an optimal position.

The combination of the previous two approaches reflects the real conditions of a headlamp not having an ideal shape.

All experiments were solved with the starting point ${\vec{s}}_{0}=\left(0,0\right)$ and a stopping point: the ideal headlamp was adjusted using general transformations and a stopping point of *ε* = 10^−6^; the real headlamp was adjusted according to the kinematic model and a stopping point of *ε* = 10^−3^; the ideal headlamp was adjusted using a penalty coefficient *ρ* = 10^12^ and a stopping point of *ε* = 10^−2^. These parameters were tuned according to the type of adjustment. These stopping point values are sufficient due to the accuracy achieved in the calibration screw settings by the industrial robot performing the adjustment.

### Ideal headlamp adjusted according to the kinematic model

For the numerical experiment, we set $\vec{s}=\left(0.4,0.1\right)$ and transformed all points according to (11). The aim was to determine the screw settings that aligned the model into an ideal position.

We then applied both algorithms and compared the results. The gradient method produced the output ${\vec{s}}_{\text{out}}^{\text{grad}}=\left(-0.414042806,-0.098633275\right)$, and the BFGS algorithm returned the vector ${\vec{s}}_{\text{out}}^{\text{BFGS}}=\left(-0.414042804,-0.098633323\right)$.

With a precision set to 10^−6^ in both algorithms, the number of decimal places was raised accordingly. Both results corresponded to the expected solution.

**Fig 6:**
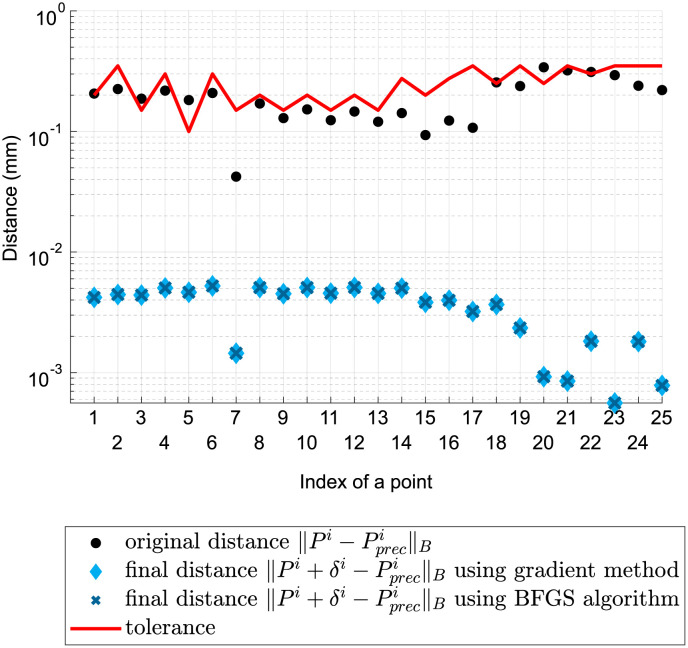
Ideal headlamp adjusted according to the kinematic model with $\vec{s}=\left(0.4,0.1\right)$—approach distances between the test points and their prescribed positions before and after optimization.

We are interested in comparing the distances between the test points and their prescribed positions before and after adjustment. Fig 6 compares the ‖.‖_*B*_ norm with the given tolerance, which is indicated by a red line. Some points in the initial position fell outside the given tolerance. By contrast, after optimization, the headlamp fully satisfied the specified conditions, and distances were reduced significantly. Namely, the mean of the distance before adjustment was 1.91⋅10^−1^, and the mean of the final distance was 3.50⋅10^−3^. The value of the cost function at the beginning of the optimization was 4.34. Both algorithms reduced the value to 8.01⋅10^−4^.

**Fig 7:**
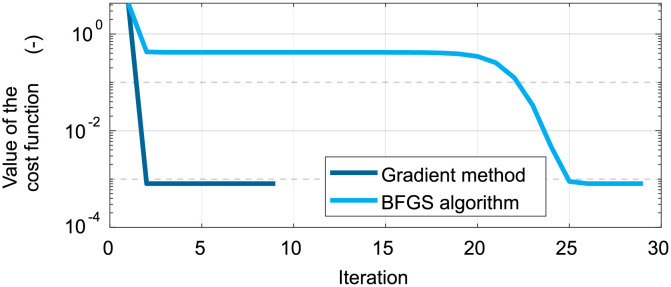
Ideal headlamp adjusted according to the kinematic model with $\vec{s}=\left(0.4,0.1\right)$—convergence history of both algorithms.

We are also interested in the performance of the algorithms. Fig 7 indicates that the gradient method needed significantly fewer iterations to reduce the cost function. However, the Golden Section method applied in this algorithm is more time-consuming.

The results in Table 2 give us a better idea of the behaviour of the algorithms and various configurations for the calibration screws.

**Table 2:** Ideal headlamp adjusted according to the kinematic model for different values of $\vec{s}$—results for both algorithms.

	Gradient method		BFGS algorithm	
$\vec{s}$	it	${\vec{s}}_{\text{out}}$	it	${\vec{s}}_{\text{out}}$
(−0.4, −0.4)	23	(0.37837, 0.35932)	29	(0.37838, 0.35933)
(−0.4, −0.2)	12	(0.38439, 0.19254)	31	(0.38439, 0.19254)
(−0.4, 0.2)	15	(0, 38498, −0, 20823)	33	(0.38499, −0.20824)
(−0.4, 0.4)	49	(0, 37698, −0, 44491)	26	(0.37698, −0.44492)
(−0.2, −0.4)	34	(0, 18865, 0, 35682)	33	(0.18865, 0.35682)
(−0.2, −0.2)	40	(0, 19440, 0, 18973)	30	(0.19441, 0.18974)
(−0.2, 0.2)	26	(0, 19448, −0, 21115)	7	(0.19449, −0.21115)
(−0.2, 0.4)	54	(0, 18626, −0, 44776)	8	(0.18627, −0.44777)
(0.2, −0.4)	14	(−0, 21026, 0, 35622)	7	(−0.21026, 0.35623)
(0.2, −0.2)	23	(−0, 20508, 0, 18923)	6	(−0.20509, 0.18923)
(0.2, 0.2)	43	(−0, 20602, −0, 21040)	27	(−0.20602, −0.21041)
(0.2, 0.4)	45	(−0, 21464, −0, 44614)	30	(−0.21465, −0.44614)
(0.4, −0.4)	20	(−0, 41959, 0, 35807)	34	(−0.41959, 0.35807)
(0.4, −0.2)	20	(−0, 41470, 0, 19144)	30	(−0.41471, 0.19145)
(0.4, 0.2)	17	(−0, 41618, −0, 20690)	31	(−0.41619, −0.20691)
(0.4, 0.4)	34	(−0, 42501, −0, 44188)	31	(−0.42502, −0.20690)

### Real headlamp adjusted according to the kinematic model

In this case, $\vec{s}=\left(0.4,0.1\right)$ again and all points were transformed according to (11). We then slightly deformed the test points, resulting in the initial model that we want to adjust and return to the prescribed position.

First, imprecise manufacturing was simulated by adding noise to all test points. Initially, several points fell outside the given tolerance, but after optimization, all points were completely within tolerance. The mean of the distance before adjustment was 1.92 ⋅ 10^−1^, and the mean of the final distance was 1.33 ⋅ 10^−2^. The value of the cost function was reduced from 4.33 to 1.39 ⋅ 10^−2^.

The next experiment simulated an error created by imprecision in the gluing process. For this simulation, we adjusted all points located on the glass 0.01 mm in the *z* direction. This adjustment caused several points to fall outside the given tolerance, but after optimization all test points completely satisfied the tolerance. The mean of the distance before adjustment was 1.92 ⋅ 10^−1^, and the mean of the final distance was 9.40 ⋅ 10^−3^. The value of the cost function was reduced from 4.36 to 5.57 ⋅ 10^−3^.

**Fig 8:**
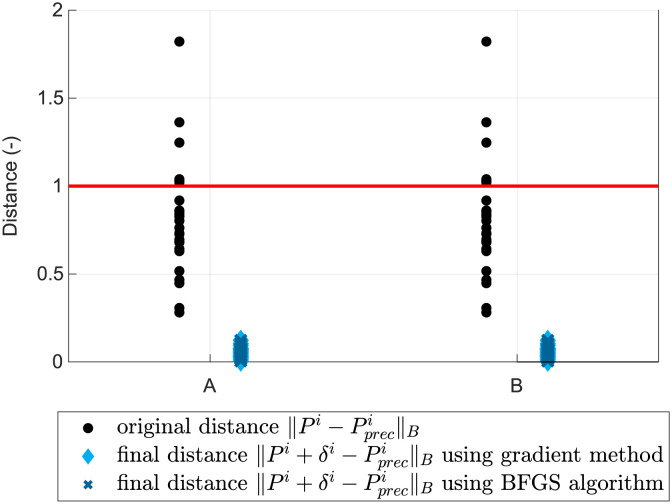
Distances between test points and their prescribed positions before and after optimization: A—poorly manufactured, B—poorly glued.

Fig 8 indicates the distances ‖.‖_*B*_ before and after transformation in relation to the given tolerance, shown as a red line for both of the experiments above. Since the given tolerance for each test point was different, we scaled all tolerances to one and scaled the distances in the same manner. We observed a significant reduction in the distance of the test points from their prescribed positions after adjustment.

### Ideal headlamp adjusted using general transformations

In this case, an ideal headlamp was modified with general shifts and rotations. This approach reflects practical situations more realistically and tested the robustness of the algorithms.

First, a rotation of 10^−2^*π* on the *x* axis was applied. The aim is to find a position for the calibration screws which compensates the rotation. The mean of the distance before adjustment was 5.14⋅10^−1^, and the mean of the final distance was 2.55⋅10^−1^. The value of the cost function was reduced from 12.03 to 4.67.

Consequently, the rotation according to the *y* axis by 10^−2^*π* was applied. The mean of the distance before adjustment was 3.71⋅10^−1^, and the mean of the final distance was 2.15⋅10^−1^. The value of the cost function was reduced from 8.59 to 4.52.

**Fig 9:**
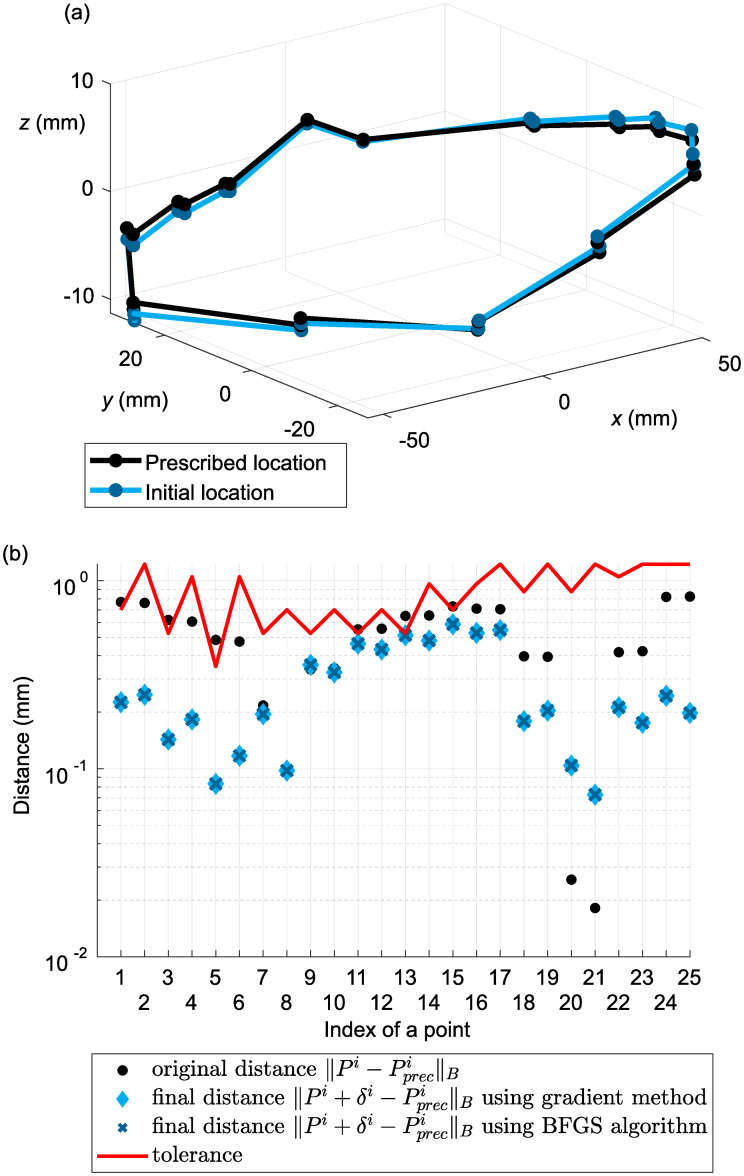
Ideal headlamp rotated around the *x* and *y* axes by 10^−2^*π*: (a) 3D location of test points; (b) approach distances between test points and their prescribed positions before and after optimization.

We then rotated the model around the *x* and *y* axes by 10^−2^*π*. Fig 9 illustrates the effect of both rotations and the distances ‖.‖_*B*_. The mean of the distance before adjustment was 5.03⋅10^−1^, and the mean of the final distance was 2.52⋅10^−1^. The value of the cost function was reduced from 9.32 to 5.95.

The model is very sensitive to rotation and it is easy to violate the mathematical conditions. However, both algorithms were able to adjust all the test points within the given tolerance.

Let us now focus only on shift along the vector (0.3, 0.3, 0.5) mm. The mean of the distance before adjustment was 5.09⋅10^−1^, and the mean of the final distance was 3.61⋅10^−1^. The value of the cost function was reduced from 8.71 to 5.98.

Finally, we combine rotation and shift. We take both angles equal to $\frac{1}{3}\cdot 10^{-2}\pi$ and the vector (0.05, 0.05, 0.05) mm. The mean of the distance at the start was 5.09⋅10^−1^, and the mean of the final distance was 2.47⋅10^−1^. The value of the cost function was reduced from 9.41 to 4.95.

**Fig 10:**
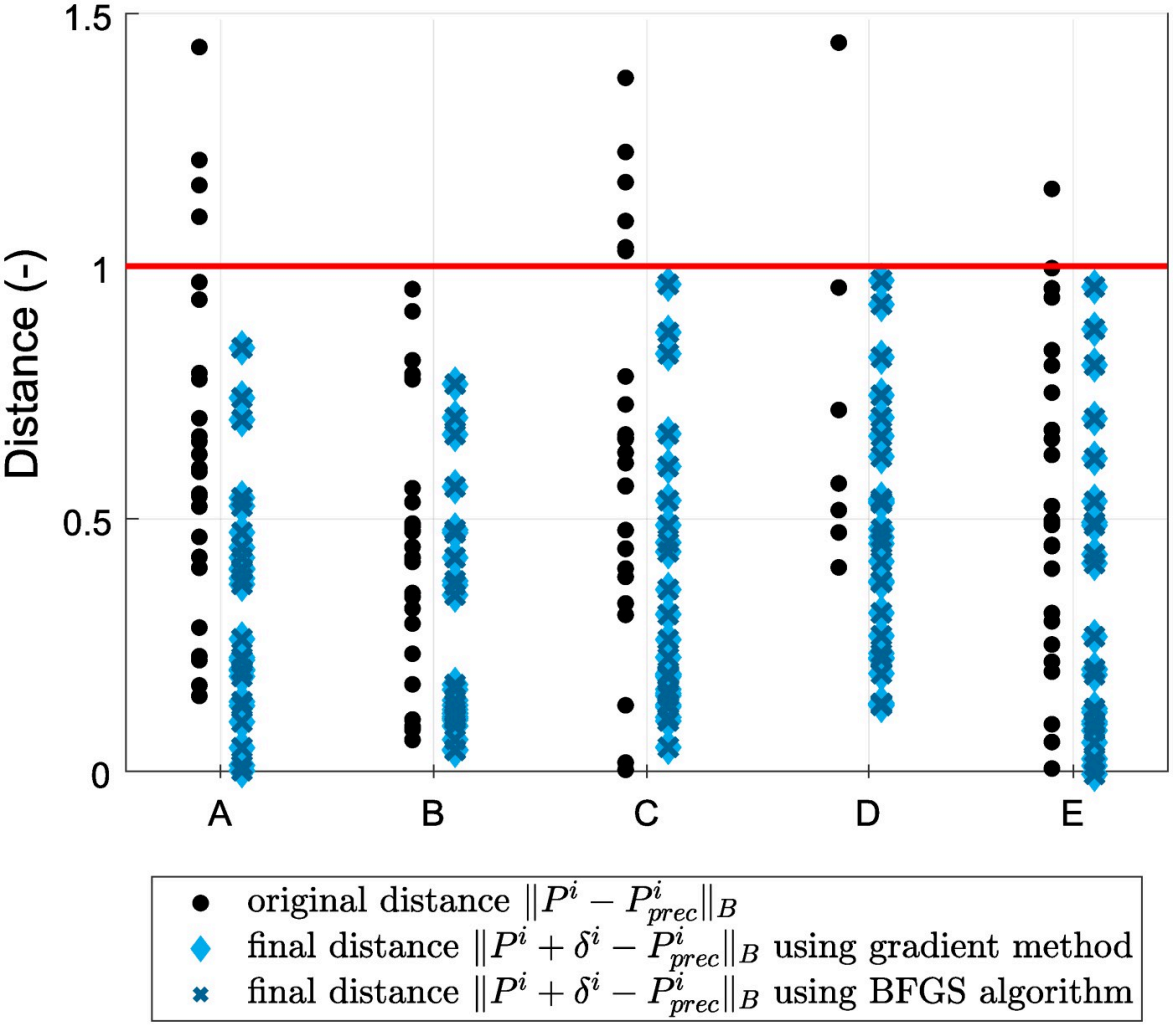
Distances between test points and their prescribed positions before and after optimization in the experiments: A—rotation about the *x* axis, B—rotation about the *y* axis, C—rotation about the *x* and *y* axes, D—shift, and E—rotation about the *x* and *y* axes and a shift.

Fig 10 shows the distances ‖.‖_*B*_ before and after the transformation in relation to the given tolerance, which is indicated with a red line for all five experiments above. Since the given tolerance for each test point was different, we scaled all tolerances to one and scaled the distances in the same manner. We observed a reduction in the distance of the test points from their prescribed positions after adjustment.

### Comparison to manual calibration by an operator

The proposed method has been successfully applied to the production line of an automotive manufacturer. In this section, we compare the proposed method (individual calibration) to manual calibration by an operator (non-individual calibration).

In manual calibration, when production commences, the first headlamp produced is taken and precisely measured using a CMM. An operator then manually and expertly estimates the optimal calibration screw setting. All subsequent headlamps adjusted on the same day are considered as having the same geometry, shape, and initial position as the reference headlamp. The same calibration screw setting is therefore used for every product produced on that day. The calibration procedure itself is non-individual and done by the operator using an industrial robot.

The proposed method achieves a major improvement in the success rate of adjustment. It is able to respond to the changes in shape and position and adjust settings accordingly. A minor disadvantage is the additional time needed for measurement and the adjustment procedure. Each headlamp on the line is checked, measured and adjusted, and compared to manual adjustment, the positions of the calibration screws can be computed very precisely for each part.

The products are considered slightly deformed and adjusted along the vector *t* = (0.1, 0.1, 0.2). Using the optimization algorithm, the optimal position ${\vec{s}}_{opt}$ of the calibration screws is computed. To test the system, we used a set of 20 test samples with random noise added to each point and applied the vector *t*. To simulate the original method, all samples were adjusted according to the same calibration screw setting ${\vec{s}}_{opt}$. Table 3 shows the results for both approaches obtained by the computer simulation and the original position before adjustment. The reference product is highlighted in grey.

**Table 3:** Comparison of results obtained by computer simulation of individual and non-individual calibration.

	No calib.		Non-ind. calib.		Individual calib.		
Sample	MD	PIT	MD	PIT	MD	PIT	Opt. Conf.
ref.	0.220	14	0.087	25	0.089	25	(−0.240, −0.168)
1	0.223	14	0.085	24	0.078	25	(−0.252, −0.215)
2	0.223	14	0.085	23	0.080	25	(−0.256, −0.187)
3	0.223	14	0.085	24	0.086	25	(−0.242, −0.187)
4	0.224	14	0.086	23	0.085	25	(−0.250, −0.181)
5	0.229	14	0.091	21	0.076	25	(−0.283, −0.286)
6	0.228	14	0.090	23	0.074	25	(−0.273, −0.292)
7	0.226	14	0.088	23	0.075	25	(−0.269, −0.301)
8	0.227	14	0.089	22	0.081	25	(−0.271, −0.179)
9	0.226	14	0.088	23	0.087	25	(−0.245, −0.188)
10	0.224	14	0.086	24	0.082	25	(−0.263, −0.167)
11	0.225	14	0.087	22	0.074	25	(−0.272, −0.212)
12	0.224	14	0.086	23	0.073	25	(−0.277, −0.253)
13	0.220	14	0.082	24	0.083	25	(−0.245, −0.182)
14	0.224	14	0.086	24	0.075	25	(−0.256, −0.278)
15	0.228	14	0.090	22	0.080	25	(−0.272, −0.191)
16	0.221	14	0.083	25	0.079	25	(−0.260, −0.174)
17	0.220	14	0.083	25	0.092	25	(−0.220, −0.187)
18	0.227	14	0.089	23	0.086	25	(−0.243, −0.205)
19	0.223	14	0.085	24	0.086	25	(−0.243, −0.182)
20	0.219	14	0.082	25	0.085	25	(−0.247, −0.162)

**Fig 11:**
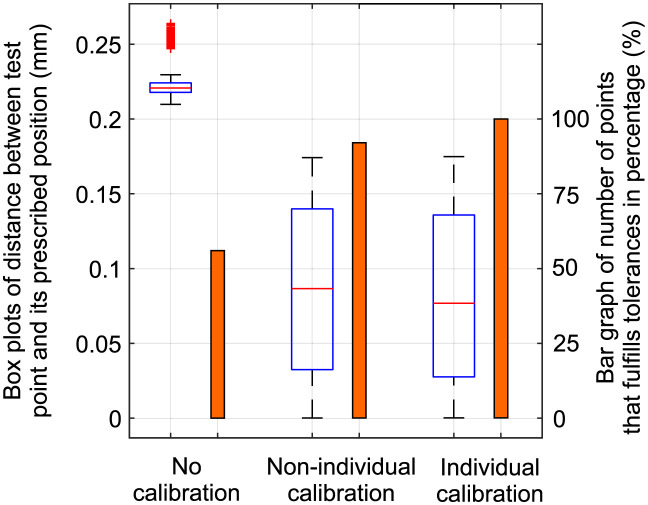
Distances between test points and their prescribed positions. The success rate of test point alignment for each headlamp.

Opt. Conf.—Optimal configuration of screws, Individual calib.—Results for individual calibration (the proposed method), Non-ind. calib.—Results of the non-individual calibration (previous method), No calib.—No calibration, MD—Mean distance, PIT—Points in tolerances,

Fig 11 compares non-individual and individual calibration with non-calibrated samples. Headlamps with no calibration indicate the highest inaccuracies and only 56% are within tolerances. Non-individual calibration produced better results with 92% of samples within tolerances. All individually calibrated samples were within tolerances and obtained slightly smaller distances.

### Comparison of digital twin with the manufacturing process

**Fig 12:**
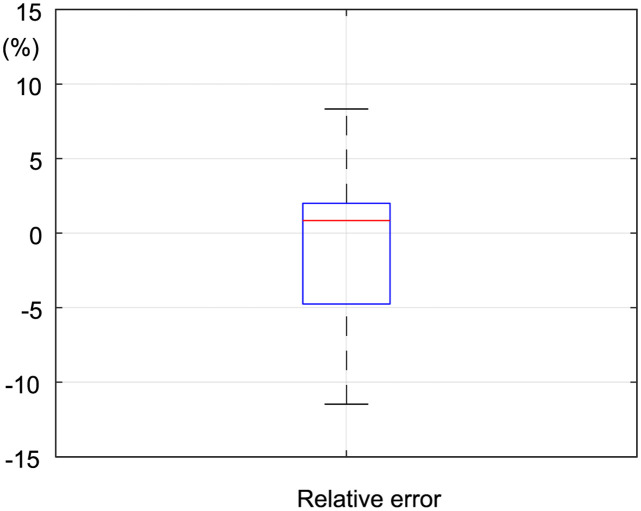
Relative error between the estimated tolerance and experimental measurement in relation to the tolerance value.

To evaluate the digital twin, we compared the predicted adjustment according to the digital twin and the results obtained from a practical run. The evaluation procedure differs from the manufacturing process in that a part is measured only once, specifically before calibration. This is due to the strict requirements in the machine cycle where post-calibration measurement cannot be performed. Fig 12 indicates the relative error between estimated tolerances according to the digital twin and experimental measurements by the CMM. Relative error relates to the maximum tolerance at each point, calculated as follows:

$$\begin{array}{c} {\text{Err}}_{\text{REL}}=\frac{X_{\text{EST}}-X_{\text{CMM}}}{\text{Toler}}, \end{array}$$ (40)

where ${\text{Err}}_{\text{REL}}$ is the relative error, $X_{\text{EST}}$ is the tolerance estimated from the digital twin, $X_{\text{CMM}}$ is the tolerance measured by the CMM, and Toler is the maximum tolerance permitted for a particular point. Fig 12 indicates that the relative error does not exceed 12%, which means, for example, all points with a tolerance of 1 mm have a maximum absolute error of 0.12 mm.

Table 4 gives a breakdown of the digital twin’s success in adjusting a specific headlamp type in a practical industrial scenario. The source data set were obtained from an industrial partner and represent the results of the digital twin calibration process performed directly on a manufacturing line. The dataset describes 84,055 fully measured headlamps before calibration and the number of points outside tolerances after calibration according to the digital twin.

**Table 4:** Success rate analysis of the practical industrial application of a digital twin in executing adjustments on a specific headlamp type.

Input			Output		
Points outside tolerances	Number of headlamps	Percentage of total	Points outside tolerances	Number of headlamps	Percentage of category
**Total**					
0–25	84,055	100.00%	0	82,532	98.19%
			1–4	1,338	1.59%
			5–10	168	0.20%
			11–25	17	0.02%
**Categorized**					
0	12,095	14.39%	0	12,087	99.93%
			1–4	8	0.07%
			5–10	0	0.00%
			11–25	0	0.00%
1–4	45,194	53.77%	0	44,567	98.61%
			1–4	619	1.37%
			5–10	8	0.02%
			11–25	0	0.00%
5–10	25,663	30.53%	0	24,975	97.32%
			1–4	597	2.33%
			5–10	88	0.34%
			11–25	3	0.01%
11–25	1,103	1.31%	0	903	81.87%
			1–4	114	10.34%
			5–10	72	6.53%
			11–25	14	1.27%

Headlamps with no points outside tolerances satisfied the limits for accuracy. The remainder of the samples were separated according to the number of points outside tolerances: low (1–4), medium (5–10) and high (11–25).

From 84,055 fully calibrated samples, 98.19% and most of the remaining (1.59%) samples were produced with few inaccuracies. A breakdown is given for the input samples according to four inaccuracy levels. Input samples at the zero (0) level indicated no points outside tolerances, representing 14.39% of all production; these were adjusted simply to improve their geometrical properties. After calibration, almost all of these samples showed zero inaccuracies, only eight samples indicating a few points outside tolerances. Input samples at the next level (1–4) represented 53.77% of production and contained a small number of inaccuracies. After calibration, 98.61% of these samples showed no points outside tolerances. The majority of the remainder had few inaccuracies (low), and eight samples had slightly more (medium). At the next level (5–10), input samples represented 30.53% of production and contained significant numbers of inaccuracies. The calibration process improved these samples to 97.32% with no points outside tolerances. Input samples at the (high) level of inaccuracy represented a minor portion of production (1.31%); the calibration procedure in this case was able to fully calibrated 81.87% of samples.

## Discussion

Several interesting topics can be discussed, the first being the optimization methods introduced in the article. The industrial partner required quick computation and high accuracy, and to investigate, we selected a first-order method (gradient method) and quasi-Newton method (BFGS algorithm). The experiments revealed that the BFGS algorithm is quicker than the gradient method.

Compared to the zero-order method, it is necessary to evaluate the gradient of the cost function. However, the proposed methods required significantly less time to find a solution. Theoretically, second-order methods converge even more quickly than quasi-Newton methods, but unfortunately, it is very difficult to compute the Hessian in every iteration.

Table 5 provides a general overview of the proposed approach compared to two state-of-the-art methods. Both of the state-of-the-art methods are more versatile than the proposed solution, however they permit only basic transformations (translation and rotation) and do not include any additional constraints. The proposed method can be applied to a specific product only, but it incorporates more complex transformations (with calibrations screws), includes constraint points, and allows a priority and limits to be set for each test point. Moreover, the proposed method has a guaranteed convergence and shorter computational time: computation speed was one of the industrial partner’s main requirements.

**Table 5:** Comparison of the results to state-of-the-art studies.

Algorithm	Description	Advantages and limitations
**Montavon, Dahlem, Schmitt [15]**		
system of equations, method using pseudo-inverse	direct methods, geometric error in each point treated separately	+ direct method + points are aligned evenly − all points have the same priority
**García, Ortega, García, Martínez [30]**		
system of nonlinear equations, fuzzy control algorithm	more complex method, positions of the points are measured by multiple sensors	+ multiple sensors + includes position errors of sensors − all points have the same priority − only basic transformations − unconstrained problem
**Proposed algorithm**		
least square method, optimization using the gradient method or BFGS algorithm	constrained opt., allows to specify tolerances for each point	+ short computation time + more complex model (with constraint points, etc.) + more complex transformations (adjustment with calibration screws, etc.) + constrained optimization + each point has its own priority − necessary to compute the gradient of the cost function − used only for a specific type of problem

Finally, we outline possible future modifications to the proposed automated product adjustment and calibration screw method. Thus far, we have worked with the assumption that the product behaves as a rigid body and that no friction exists between the calibration screws and the product. We also regard the problem as static and independent of time. The adjustment process does not depend on the screwing sequence. In the future, it may be necessary to modify the kinematic model to describe a product as an elastic body. In this case, the kinematic model will require modification and use finite element methods.

Another possible modification is to consider the friction between the calibration screws and the product according to the Coulomb model of friction, for example. In this article, we neglected friction. If we consider friction, both the kinematic model and optimization algorithm would need to be redesigned since the cost function would no longer be continuously differentiable, only Lipschitz continuous. For this reason, a method which is suitable for minimizing a function that is not differentiable should be used. These types of method are called non-smooth methods; one of the most commonly applied is the bundle method. A detailed discussion about using the bundle method for this type of problem is in [31, 32]. It may also be necessary to model the adjustment process as dependent on time so that the correct screwing sequence is considered and maintained.

## Conclusion

The article introduced a digital twin model for automated product adjustment using calibration screws. The automated product adjustment procedure was designed to find the optimal configuration for a set of calibration screws to minimize the distances between the test points and their prescribed positions and therefore eliminate geometric error. The proposed strategy consists of solving two sub-problems: the design of a digital twin for a headlamp and optimization using calibration screws.

We formulated an optimal product adjustment procedure for minimizing the locally Lipschitz continuous cost function, which in this case is continuously differentiable and subject to inequality constraints. To solve the optimization problem, we applied the gradient method and BFGS algorithm.

The body of knowledge presented in the article contains a novel strategy for precisely manufacturing headlamps using a digital twin. Headlamps are equipped with compensatory elements that can adjust the fixing points of the product. Before we applied the proposed automated adjustment method, only one headlamp per day was measured and adjusted manually by a machine operator. The remaining products were adjusted according to the same settings throughout the day by a machine. The main advantage of the proposed method is its ability to adjust each part with individual settings and thereby reduce production variability. Its second advantage is in process capability: the manufactured part is measured during the adjustment process, yielding final tolerances. This feature has an enormous impact on the Process Capability Index. Tolerances only need to meet the customer’s specification limits instead of the stricter tolerances applied in the SPC strategy.

The results showed that the novel method was able to align all headlamps, whereas non-individual calibration is able to aligns only 92% of parts. The digital twin method was applied to 84,055 headlamps samples, yielding successfully alignment by the calibration machine in 98.19% of samples.

## Supporting information

S1 Data (ZIP)
